# A cross-national and longitudinal analysis of handwashing and its predictors during the COVID-19 pandemic in France and Belgium

**DOI:** 10.1080/21642850.2022.2120882

**Published:** 2022-09-15

**Authors:** Mathias Schmitz, Robin Wollast, Alix Bigot, Olivier Luminet

**Affiliations:** a Institute for Research in the Psychological Sciences, Université catholique de Louvain, Louvain-la-Neuve, Belgium; b Fund for Scientific Research (FRS-FNRS), Brussels, Belgium

**Keywords:** Handwashing, health behavior, COVID-19, pandemic, TPB

## Abstract

Background: Using a longitudinal design, we investigate how the adherence to handwashing and its underlying socio-psychological predictors evolved over time during the COVID-19 pandemic and under distinct circumstances (e.g. when the crisis was more acute or chronic). Method: We collected data (*N* = 753) in Belgium and France at the onset of the COVID-19 pandemic when the crisis was at its peak (April 2020), and almost a year later (February 2021), when the outbreak was more manageable. Results: Regression models suggest that the compliance with handwashing and its pattern of underlying predictors remained remarkably stable over time despite the variations in contextual factors such as the severity of the health crisis and the stringency of health measures. As such, the findings also highlight the robustness of the models that predict it, namely the Theory of Planned Behavior. The intentions to perform the behavior, the perceived control over it, and being part of the (para)medical field were among the strongest predictors. Conclusions: In practice, the stability of the underlying factors suggests a set of action levers that can be used in communication campaigns aimed at fostering its adherence throughout the pandemic.

Since March 2020, the world has been plunged into a health crisis caused by the SARS-CoV-2 virus. To surmount the COVID-19 pandemic, governments worldwide began an unprecedent effort to implement a series of preventive behaviors to stop the spread of the virus as recommended by the World Health Organization (WHO, 2021a). Measures such as handwashing, social contacts limitation, physical distancing, and mask wearing have been shown to play a critical role in confining the circulation of the virus among the population (e.g. Brauner et al., 2021; Talic et al., 2021). Despite their imperative, the adoption of these protective measures has proven to be an arduous task. Therefore, identifying the socio-psychological factors that may foster or hamper their application is vital to overcome the pandemic (Van Bavel et al., 2020). Here we focus specifically on handwashing behavior, which is one of the most frequently recommended health behavior during health crisis situations. Although previous research has examined such factors during the COVID-19 crisis (e.g. Bigot et al., 2021; Wollast et al., 2021), most were cross-sectional and little is known about their predictive stability over long time periods and under distinct circumstances (e.g. the severity of the crisis). The aim of the present research was therefore to investigate through a longitudinal design across two different countries (France and Belgium), the socio-psychological predictors of handwashing at two distinct time points: At the beginning of the pandemic when the situation was new and acute (March 2020), and almost a year later when the crisis entered a more chronic phase (February 2021).

## Timeline of the pandemic

By early March 2020, all European countries had confirmed COVID-19 cases (Reuters, 2020), highlighting the rapid spread of the virus across the population. The situation quickly became critical given the lack of preparedness; for instance, there was a worldwide shortage of face masks (WHO, 2021b). Governments quickly responded with drastic measures as incited by the WHO: all non-essential activities were shut down, and citizens were mandated to stay at home (i.e. lockdown).

**Figure 1.**
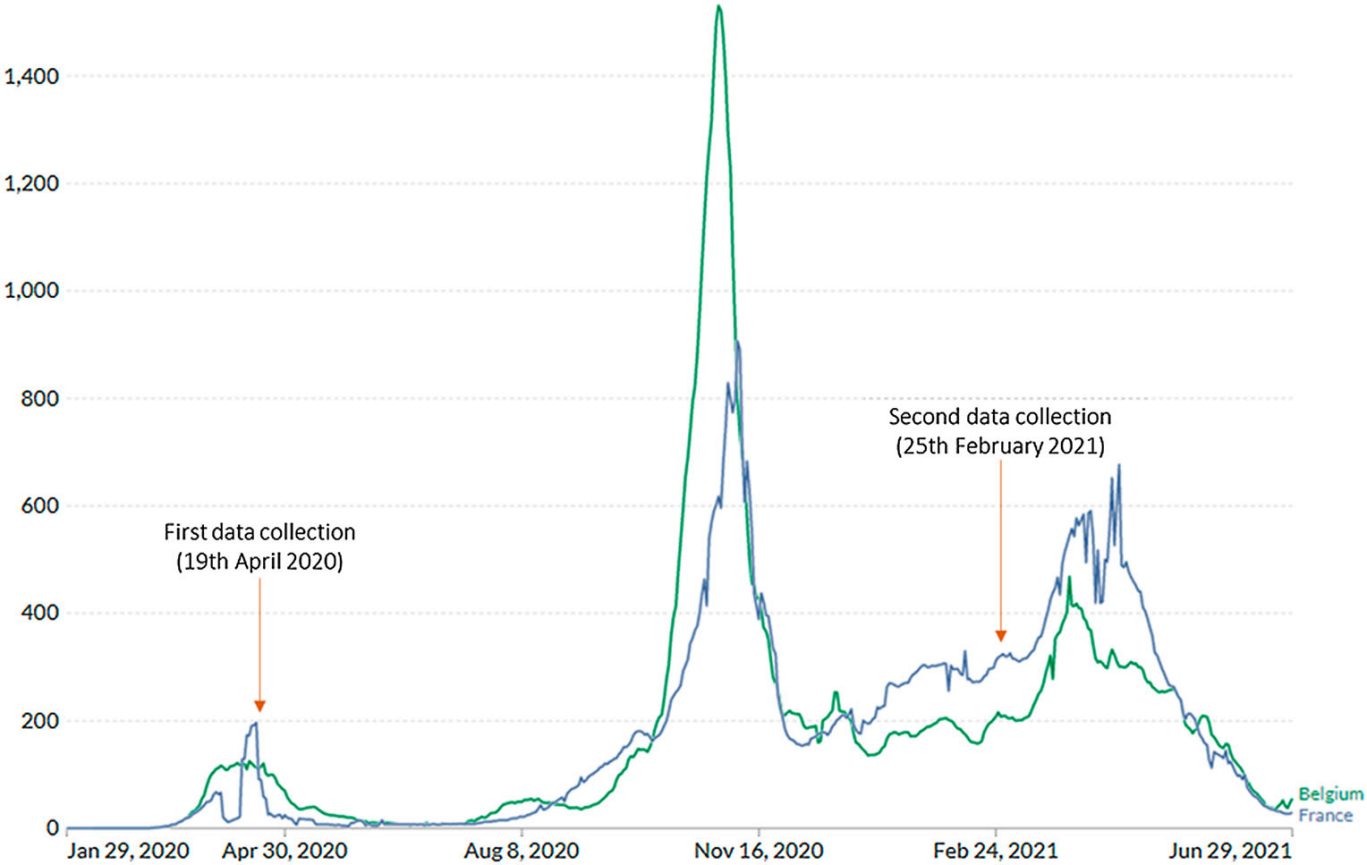
Daily new confirmed COVID-19 cases per million people (7-day rolling average). Source: adapted from Our World in Data (2021).

**Figure 2.**
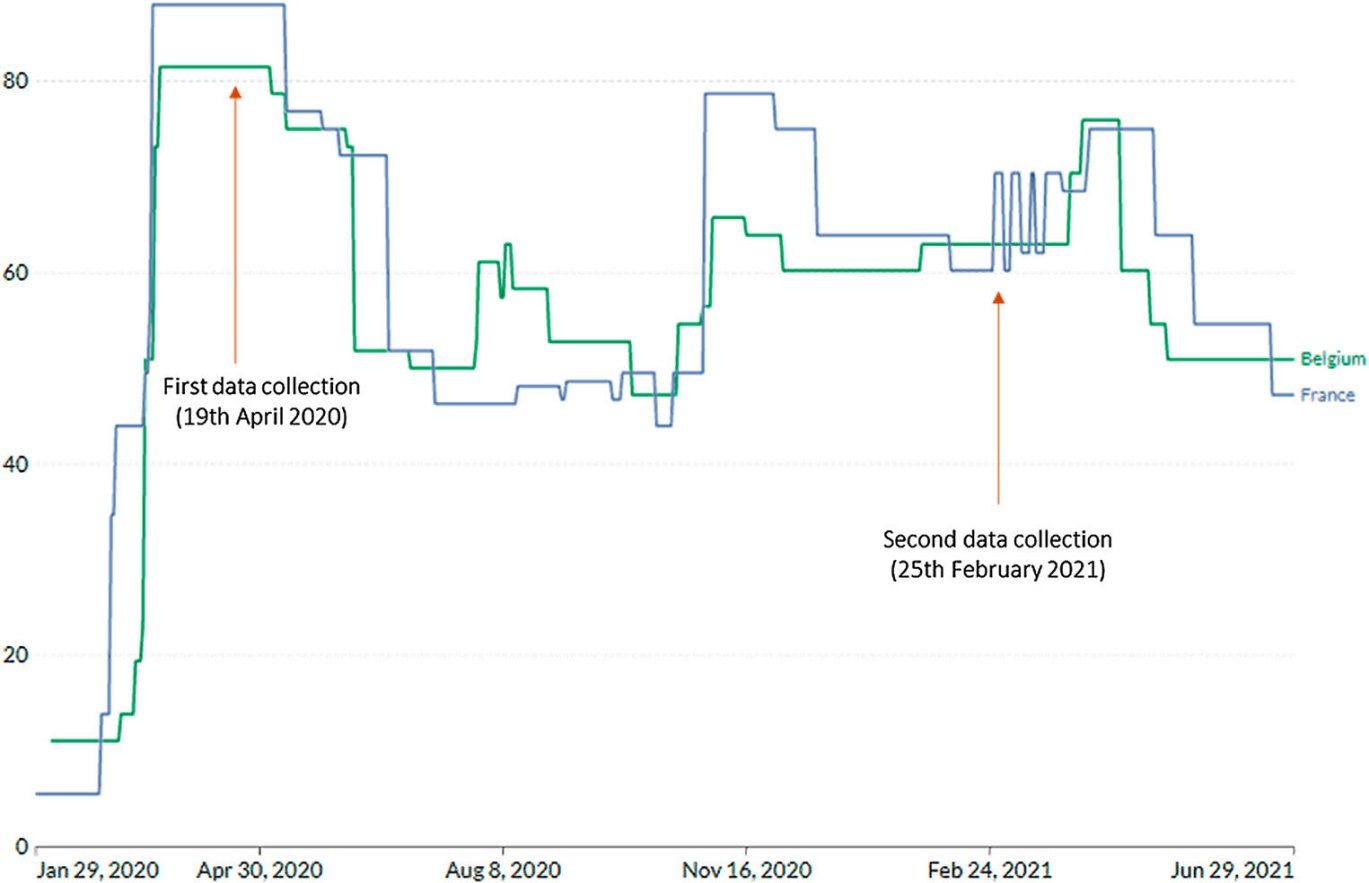
COVID-19: Stringency index. Note: The stringency index is a composite measure based on nine response indicators including school closures, workplace closures, and travel bans, rescaled to a value from 0 to 100 (100 = strictest). If policies vary at the subnational level, the index shows the response level of the strictest subregion. Source: Adapted from Our World in Data (2021) and Hale et al. (2021).

The first lockdown measures started on the 16th of March in France, and the 17th in Belgium, which corresponds to the beginning of the first ‘wave’ of coronavirus cases. Although the restriction measures were gradually relaxed by the end of the first wave (by early June for France, and early May for Belgium), they were never completely abolished and had to be strengthened again as the number of cases rose at each wave (see Figure 1 for the number of cases and Figure 2 for the severity of the measures). Moreover, new measures were introduced as the pandemic unfolded (e.g. face masks became mandatory and a COVID-19 safe pass was implemented).

Both countries simultaneously experienced the first two waves of COVID-19 cases, with the peak being respectively mid-May 2020 and early November 2020. The first two waves were the most devastating given the lack of medical supplies (e.g. face masks) and vaccine availability by that time (Our World in Data, 2022). Vaccination campaigns began in January 2021, which helped reduce the spread of the virus and the overburden of the medical sector (Liu et al., 2021). Nonetheless, health measures had to be maintained (or even strengthened) as new waves of COVID-19 cases kept emerging. Moreover, the advent of these new cases was fostered by a reduction in compliance with the measures and vaccination intention during the pandemic as they were undermined by the spread of conspiracy theories and the lack of trust in the government or health institutions (e.g. Pavela Banai et al., 2021; Van Oost et al., 2022).

## Stopping the spread of the virus with health behaviors

The SARS-CoV-2 virus is transmissible by air through inhalation or by contact with contaminated surfaces (Center for Disease Control and Prevention – CDC, 2020). Therefore, washing hands has proven to be a crucial health behavior to mitigate infections (Brauner et al., 2021; Saunders-Hastings et al., 2017; West et al., 2020). Consequently, it (along with other preventive guidelines such as mask wearing, social contacts limitation, and physical distancing) was fostered by the health institutions (e.g. WHO, CDC) and mandated by the authorities since the commencement of the outbreak. Contrary to other health behaviors recommended during the pandemic that acted against basic human needs such as autonomy or relatedness (e.g. social contacts limitation), handwashing is considered as a classical health behavior, which is frequently promoted to limit the spreading of infectious diseases. It can thus be considered as a habit for most of the population, even before the onset of the pandemic. Moreover, sanitizing hands is relatively easy to perform (i.e. does not necessarily require a conscious effort), is part of the social norms, and most people understand its importance in terms of diseases prevention (Hagger et al., 2020).

To better understand and foster the adoption of health behaviors, it is essential to identify the underlying socio-psychological factors that may boost or hinder their acceptance. One of the most prominent models in health psychology is the Theory of Planned Behavior (TPB; Ajzen, 1991; Ajzen & Schmidt, 2020) which has been used to explain the mechanisms that drive health behaviors during the pandemic (e.g. Hagger et al., 2020; Hamilton et al., 2020). This theory holds that behaviors can be explained by four key psychological components, namely the *intentions* to perform the action, the *attitudes* they have toward the behavior (i.e. the expectations carrying out the behavior), the perceived *control* they have over it (i.e. how easy it is to put into practice), and the social norms (i.e. belief that others positively value and adhere to the behavior). In principle, the enactment of the behavior becomes more likely the higher the intentions and perceived control, the more positive the attitudes, and the stronger the subjective norms are. Therefore, we expected that a higher endorsement of the TPB components would boots adherence to handwashing.

In practice, and of particular interest, Bigot et al.’s (2021) study revealed that in March 2020, handwashing was positively predicted by intentions to act, positive attitudes, perceived control, whereas the subjective norms did not contribute to the facilitation of this behavior (for other studies about the TPB in the context of the COVID-19 pandemic, see Clemens et al., 2021; Kim et al., 2020; Wollast et al., 2021; Zhang et al., 2020).

Emotion-related predictors are often ignored when predicting health behaviors, although they can play a key role in their development and maintenance. For instance, feeling attentive/determined or frightened/anxious were found to be related to higher hand hygiene, together with the anxiety about one own’s health. Personality aspects such as impulsivity (i.e. lack of premeditation, urgency, sensation seeking, lack of perseverance) were also examined by Bigot et al. (2021) based on the idea that higher levels on those personality traits would potentially undermine the adherence to health behaviors given that they require planned action and perseverance. Likewise, social connection (i.e. social relationships, empathy, loneliness) elements were incorporated given that one’s social network may foster the dissemination of adherence to health behaviors (or the inverse if the network is composed of people whom are reluctant to comply with health measures), that greater empathy would facilitate adherence (e.g. empathy towards elderly people that were more vulnerable to the virus could boots handwashing to protect them), and that loneliness could undermine it given that is if often associated with poor mental health and less preoccupation for others (Kim et al., 2015; Segrin & Passalacqua, 2010). Despite these rationales, neither impulsivity nor social connection yield significant effects on handwashing when measures were taken at T1 by Bigot et al. (2021; but see also Schmitz, Wollast, et al., 2022). We decided to maintain these predictors in the present research to allow comparison with past studies and to provide an opportunity to examine their potential impact on different contexts. Although the study conducted by Bigot et al. (2021) was carried out in Belgium, other investigations (Schmitz, Wollast, et al., 2022; Wollast et al., 2021) showed that the findings held for France as well, despite the country’s more stringent measures during the pandemic (see Figure 2). In essence, these studies identified unique predictors associated with handwashing (and other health behaviors).

A noteworthy limitation of the above research effort (i.e. Bigot et al., 2021; Schmitz, Wollast, et al., 2022; Wollast et al., 2021), however, is that the findings are limited to a single time point, namely at the beginning of the outbreak (March 2020), which is a very particular period for several reasons. Indeed, little was known about the virus (e.g. transmission mechanisms, comorbidity factors, consequences of the disease), most of the health behaviors were novel to the population, and masks and vaccines were not yet available. Taken together, these circumstances bolstered the fear and anxiety among the general population (Asmundson & Taylor, 2020). The question thus remains whether the prediction pattern identified in previous studies would hold further in time and under quite different circumstances. For instance, when the situation is less critical, turned into a more chronic phase and people had time to get used to the health behaviors (or got tired of implementing them).

## The present study

The aim of the present research was to investigate if the underlying socio-psychological predictors of handwashing hold at two very distinct time points and circumstances during the COVID-19 pandemic, despite the expectation that the mean levels for handwashing adherence and the different factors predicting the health behavior would vary with time. The first data collection builds on previous work from Bigot et al.’s (2021) and corresponds to the onset of the pandemic (T1, March18−April 19, 2020), and the (new) second longitudinal data collection took place almost a year later (T2, February 25−March 14, 2021) in-between the second and third wave of COVID-19 cases. Participants from the present study participated in both T1 and T2 data collections. Importantly, and as mentioned above, the circumstances at T2 were very different from T1: the situation was less novel and acute, the measures were less stringent, people had time to get used to (or got tired of) applying the preventive behaviors. Indeed, previous psychological studies found discrepancies in predictive patterns across time during the COVID-19 pandemic (see Rudert et al., 2021).

## Method

### Participants

French-speaking participants from Belgium and France were recruited via an online survey and social media at two time points almost one-year apart (312 days). The initial sample of the first data collection (T1; March18−April 19, 2020) comprised a total of 3285 participants from Belgium and France^1^ and came from Bigot et al.’s (2021).^2^ A total of 753 (*n*_Belgium_ = 565, *n*_France_ = 188) respondents also took part in the second data collection (T2; February 25−March 14, 2021). That is, participants from T1 and T2 used in the present study were the same. The mean age was 41.42 (SD = 14.71), 77% were females, and 86% had at least completed secondary school. At T1, 88% reported that they did not have been infected by the SARS-CoV-2 virus (with or without a test), while 83% declared that they had no relatives that had been infected by the virus. Finally, 82% reported having no comorbidity factors related to the COVID-19 disease, and 73% said that they did not have a relative with health-related risk factors.

### Measures

This work is an extension of Bigot et al. (2021) in which we reused their measures to further assess how the determinants of health behaviors evolved over time and across countries. For additional information about these instruments please refer to the article. The correlations between the measures at T1 and T2 can be found in Table 1.

**Table 1.** Descriptives, comparisons, and relations between study variables at T1 and T2.

	Data collection		Wilcoxon’s *r*	*r*_(T1,T2)_
	T1	T2		
Health behavior				
Handwashing	**3.00 (2.00) [3.13 (0.94)]^a^**	**3.00 (2.00) [3.00 (0.94)]^b^**	**0**.**18**	.64***
Socio-demographics				
Sex (female)	583 (77%)^a^	583 (77%)^a^	–	1.00***
Age	**40.00 (21.00) [41.42 (14.71)]^a^**	**41.00 (22.00) [42.35 (14.69)]^b^**	**1**.**94**	**1**.00***
Education level (Secondary)	**641 (85%)^a^**	**653 (87%)^b^**	–	.78***
(Para)medical field (Yes)	229 (30%)^a^	220 (30%)^a^	–	.74***
Theory of planned behavior				
Intentions	**5.00 (1.00) [4.66 (0.66)]^a^**	**4.00 (1.00) [4.19 (0.95)]^b^**	**0**.**49**	.44***
Attitudes	**5.00 (1.00) [4.45 (0.77)]^a^**	**4.00 (1.00) [4.24 (0.91)]^b^**	**0**.**21**	.35***
Social norms	**4.00 (1.00) [3.97 (1.04)]^a^**	**3.00 (1.00) [3.29 (1.19)]^b^**	**0**.**48**	.37***
Perceived control	**5.00 (1.00) [4.37 (0.92)]^a^**	**4.00 (1.00) [4.12 (0.98)]^b^**	**0**.**28**	.45***
Emotions				
Attentive/determined	**3.60 (1.00) [3.49 (0.75)]^a^**	**3.40 (1.20) [3.35 (0.86)]^b^**	**0**.**15**	.34***
Enthusiastic/happy	**2.25 (1.25) [2.34 (0.81)]^a^**	**2.75 (1.50) [2.74 (0.85)]^b^**	**0**.**37**	.28***
Angry/agitated	**2.80 (1.40) [3.73 (0.94)]^a^**	**3.00 (1.40) [2.95 (0.95)]^b^**	**0**.**22**	.39***
Frightened/Anxious	**2.50 (1.33) [2.52 (0.85)]^a^**	**2.00 (1.17) [2.17 (0.81)]^b^**	**0**.**37**	.43***
Physiological aspects				
Health anxiety	**2.80 (1.20) [2.75 (0.76)]^a^**	**2.60 (1.00) [2.56 (0.77)]^b^**	**0**.**28**	.58***
Impulsivity				
Premeditation	4.00 (1.00) [4.00 (0.70)]^a^	4.00 (1.00) [4.00 (0.68)]^a^	0.00	.57***
Urgency	2.50 (1.50) [2.45 (1.03)]^a^	2.50 (1.00) [2.52 (1.00)]^a^	0.05	.57***
Sensation seeking	**2.00 (2.00) [2.06 (1.02)]^a^**	**2.00 (2.00) [2.25 (1.04)]^b^**	**0**.**21**	.60***
Perseverance	4.00 (1.00) [4.07 (0.90)]^a^	4.00 (1.00) [4.02 (0.90)]^a^	0.06	.60***
Social connection				
Empathy	**4.25 (0.75) [4.14 (0.62)]^a^**	**4.00 (1.00) [3.99 (0.65)]^b^**	**0**.**29**	.64***
Loneliness	**2.33 (2.00) [2.42 (1.11)]^a^**	**3.00 (2.00) [2.87 (1.17)]^b^**	**0**.**36**	.45***

Note: Descriptives are the median (interquartile range) [mean (standard deviation)] for continuous variables and frequency (percentage) for binary variables. Information in parentheses next to binary variables indicates the value set to 1 (versus 0). Different superscripts indicate significant differences on Wilcoxon’s two-sample paired signed-rank test (all *p* < .001) between T1 and T2. Significant differences are highlighted in bold. The correlation *r* corresponds to the Spearman correlation between variables at T1 and T2. ***p < .001

#### Health behavior

Handwashing was assessed by the means of a single item. Participants reported to what extent they complied with this behavior on a 5-point scale (1 = ‘never’, 2 =  ‘1–5 times a day’, 3 =  ‘6–10 times a day’, 4 =  ‘11–15 times a day’, 5 = ‘more than 15 times a day’). This measure was based on the guidelines from sanitary institutions (e.g. CDC, 2021; WHO, 2021a), that advise people to wash their hands at the very least before and after eating and after using the bathroom.

#### Theory of planned behavior components

We assessed the four components of the TPB (Ajzen, 1991) by the means of a single item by component on a 5-point Likert scale (1 = ‘totally disagree’ to 5 = ‘totally agree’). The items were the following: ‘I believe that washing hands will limit spreading of the coronavirus’ (attitudes), ‘I am ready to wash my hands’ (intentions), ‘For me, handwashing is easy’ (perceived control), and ‘My relatives expect from me to wash my hands’ (social norms).

#### Emotions

Participants indicated their current emotional states with the French Positive and Negative Affect Scale – State version (PANAS; Gaudreau et al., 2006; Watson et al., 1988) on a 5-point Likert scale (1 = ‘totally disagree’ to 5 = ‘totally agree’). As in Bigot et al. (2021), these emotions were grouped into four categories: attentive/determined (MacDonald’s *ω*_T1_ = .76, *ω*_T2_ = .86), enthusiastic/happy (*ω*_T1_ = .72, *ω*_T2_ = .83), angry/agitated (*ω*_T1_ = .78, *ω*_T2_ = .85), and fearful/anxious (*ω*_T1_ = .81, *ω*_T2_ = .83).

#### Health anxiety

To measure how anxious people were about their health we selected five items from the Whiteley Index (Pilowsky, 1967) (*ω*_T1_ = .71, *ω*_T2_ = .72). A sample item was ‘I am afraid of getting sick’ (1 = ‘totally disagree’ to 5 = ‘totally agree’).

#### Impulsivity

Six items from the French version of the UPPS impulsive behavior scale (Van der Linden et al., 2006; Whiteside et al., 2005) were selected to assess this personality trait (1 = ‘totally disagree’ to 5 = ‘totally agree’). The dimensions of premeditation (*ω*_T1_ = .68, *ω*_T2_ = .66), urgency (*α*_T1_ = .76, *ω*_T2_ = .75)^3^ were built on two items each, and the dimensions of sensation seeking, and perseverance comprised a single item each.

#### Social connection

Three dimensions were built from various scales following Bigot et al. (2021). Empathy (*ω*_T1_ = .73, *ω*_T2_ = .78) was measured by the means of four items from the Interpersonal Reactivity Index (Davis, 1983). Feeling lonely (*ω*_T1_ = .83, *ω*_T2_ = .86) was assessed with three items from a Short Scale for Measuring Loneliness in Large Surveys (Hughes et al., 2004). Three items were used to assess social relationships (*ω*_T1_ = .49, *ω*_T2_ = .58) between the participants and close others (family, friends, and neighbors) on a scale from 1 (no bond) to 5 (very strong bonds) (Morton et al., 2020). The social relationships component was not included in the current analyzes given its low reliability at both time points.

#### Demographic information

Participants provided their age, gender, level of education, country of residence, and whether their work/studies were related to the (para)medical field. Participants also indicated if they believed to have themselves or close others been infected by the SARS-CoV-2, with or without being tested. Similarly, they specified if their close others or themselves suffered from chronic diseases that could place them at risk in the current pandemic.

### Procedure

Participants from T1 were contacted through mailing list or social platforms and asked to fill-in an online Qualtrics survey that lasted about 20 minutes. At the end of the survey, participants were asked if they would be willing to take part in follow-up studies. Those that accepted to be re-contacted received an email with an invitation to an online Qualtrics survey for the follow-up study, T2. This second survey was similar to the first one, with the exception that additional scales (not analyzed here) were included at the end of the questionnaire for an independent study. Participants took about 20 minutes on average to fill-in the survey. All participants provided informed consent prior to the study. The research program was conducted in an ethical and responsible manner, in accordance with the principles stated in the Declaration of Helsinki. The project was approved by the ethical committee from the Research Institute for Psychological Sciences at Université catholique de Louvain (Project 2021-13).

### Statistical analyzes

Multiple regression models (with ordinary least squares estimations) were used to assess the impact of the predictors on handwashing. Specifically, independent linear regression models were estimated for T1 and T2 separately.^4^ Regressions’ assumptions were tested via visual inspection of diagnostic plots (e.g. Q-Q plots of residuals, Residuals vs. Fitted values; available in the Rmarkdown report located in the public repository) and no severe violations were found. We examined the continuous variables’ distribution via visual inspection of Q-Q plots (available in the public repository) which revealed that most were not normally distributed. Therefore, we rely on non-parametric statistics whenever suited (in Table 1).^5^

The data and R scripts (R version4.1.2; for more information about the packages’ version see the Rmarkdown report available in public repository) to carry out the analyzes are publicly available on the Open Science Framework repository: https://osf.io/qvd6h/?view_only=20cb4ff6cb0149a6b07b2f9670e34a57.

## Results

### Descriptives, differences, and relations between T1 and T2 measures

Descriptive statistics, comparisons tests, and relations for the study variables are presented in Table 1. As can be seen, there was a significant decrease in application of handwashing from T1 to T2, although the effect size was very small. Likewise, there was a significant decrease in all the TPB-components across time, with small (attitudes and perceived control) to medium (intentions and social norms) effect sizes.

Regarding emotions, participants reported being more attentive/determined and frightened/anxious at T1 than at T2, whereas it was the opposite for enthusiastic/happy and angry/agitated. As for the clinical aspects, there was a significant decrease in health anxiety through time, whereas there was an increase in one of the impulsivity aspects, namely sensation seeking. Social connection variables also evolved through time, such that empathy decreased, whereas feeling lonely increased. All these effect sizes were small.

### Handwashing predictions

We assessed the impact of handwashing at T1 and T2 through multiple linear regression analyzes (see Table 2).

**Table 2.** Multiple linear regression analyzes associated with the application of handwashing at T1 and T2.

	T1			T2		
	*b* (95% CI)	partial-*η^2^*	*p*	*b* (95% CI)	partial-*η^2^*	*p*
Socio-demographics						
Sex (female)	**0.18** (**0.02, 0.34)**	.**007**	.**026**	0.10 (−0.04, 0.25)	.003	.168
Age	0.02 (−0.05, 0.09)	<.001	.563	−0.02 (−0.09, 0.04)	<.001	.508
Education level (Secondary)	−0.01 (−0.18, 0.16)	<.001	.911	0.02 (−0.16, 0.19)	<.001	.851
(Para)medical field (Yes)	**0.25** (**0.12, 0.39)**	.**018**	**<.001**	**0.27** (**0.14, 0.40)**	**.022**	**<.001**
Theory of planned behavior						
Intentions	**0.26** (**0.16, 0.36)**	.**032**	**<.001**	**0.22** (**0.15, 0.30)**	**.041**	**<.001**
Attitudes	0.03 (−0.05, 0.11)	<.001	.490	0.02 (−0.05, 0.08)	<.001	.638
Social norms	0.04 (−0.03, 0.12)	.002	.255	**0.08** (**0.02, 0.14)**	**.008**	.**015**
Perceived control	**0.13** (**0.05, 0.21)**	.**014**	.**001**	**0.20** (**0.12, 0.27)**	**.035**	**<.001**
Emotions						
Attentive/determined	**0.10** (**0.02, 0.18)**	.**008**	.**015**	0.08 (−0.00, 0.17)	.005	.055
Enthusiastic/happy	−0.02 (−0.10, 0.06)	<.001	.645	0.00 (−0.09, 0.09)	<.001	.972
Angry/agitated	**0.09** (**0.01, 0.17)**	.**006**	.**034**	**0.11** (**0.03, 0.19)**	**.009**	.**009**
Frightened/anxious	0.01 (−0.07, 0.10)	<.001	.747	−0.02 (−0.10, 0.06)	<.001	.645
Physiological aspects						
Health anxiety	0.03 (−0.04, 0.10)	.001	.372	0.01 (−0.06, 0.07)	<.001	.806
Impulsivity						
Premeditation	0.03 (−0.04, 0.09)	<.001	.420	0.06 (−0.00, 0.12)	.005	.068
Urgency	0.01 (−0.05, 0.08)	<.001	.724	−0.04 (−0.11, 0.02)	.003	.155
Sensation seeking	−0.05 (−0.12, 0.02)	.003	.174	−0.03 (−0.10, 0.03)	.002	.286
Perseverance	0.04 (−0.02, 0.11)	.002	.195	0.02 (−0.04, 0.08)	<.001	.580
Social connection						
Empathy	0.04 (−0.02, 0.11)	.002	.220	0.00 (−0.06, 0.06)	<.001	.902
Loneliness	−0.06 (−0.14, 0.01)	.004	.080	0.01 (−0.06, 0.09)	<.001	.704

Note: Significant effects (*p* < .05) are highlighted in bold. Continuous predictors were standardized. Information in parentheses next to binary variables indicates the value set to 1 (versus 0).

Regarding the socio-demographic variables, being female and being part of the (para)medical field both had a positive impact on handwashing at T1, although only the latter reached significance at T2. In terms of the TPB components, positive intentions and a higher perceived control over the behavior increased hand hygiene at T1. This was also the case at T2, with the addition of a positive effect from the social norms. Regarding emotion predictors, being more attentive/determined and angry/agitated fostered the application of the health behavior at T1, but only the former reached significance at T2. Across both time points, TPB-intentions presented the largest effect size, followed by being part of the (para)medical field at T1, and perceived control for T2. A similar pattern of results for the T2 regression model (see the Rmarkdown report from the public repository) was found when controlling for handwashing at T1, although the predictors’ effect sizes were smaller given the large shared variance between the behavioral measures at the two time points.

## Discussion

Previous research (Bigot et al., 2021; Schmitz, Wollast, et al., 2022; Wollast et al., 2021) has identified underlying socio-psychological factors associated with the adoption of preventive health behaviors, which are paramount to overcome the COVID-19 pandemic (Van Bavel et al., 2020). A noticeable shortcoming, however, is that these studies were carried out during the onset of the COVID-19 pandemic. This peculiar period was characterized by high levels of uncertainty, fear, anxiety, and novelty regarding some of the preventive behaviors (Asmundson & Taylor, 2020). On this basis and following the recommendations from Rudert et al. (2021), the aim of the current research was to investigate whether the level of adherence and the prediction pattern would hold or evolve over long time period and under very distinct circumstances. To do so, we relied on longitudinal samples from France and Belgium collected at two time points, namely at the beginning of the outbreak (T1, March 2020), and almost a year later when the crisis was less severe (T2, February 2021).

A first finding reveals a very small decrease (Wilcoxon’s *r* = 0.18) in the adherence to handwashing between the first and second data collection. These results are consistent with other data collected in Belgium (Motivation Barometer, 2021; Sciensano, 2021). They correspond with the idea that the repetition of a behavior over such a long period tends to create a habit, which renders the behavior less effortful, more automatic, and thus more sustainable (Hagger et al., 2020; Hagger & Hamilton, 2020). This slight decline in adherence may be explained in part by a diminution in all the TPB-components (intentions, attitudes, social norms, and perceived control) which foster enactment (see Ajzen, 1991).

Several aspects seem to suggest that handwashing is a robust behavior that can be relatively well maintained over long time periods and under very distinct circumstances. Indeed, the level of adherence remained stable despite the fact that the development of habits usually requires a stable context (Wood, 2017), which was clearly not the case in these troubled times. In the same vein, people kept practicing hand sanitizing even though internal and external incentives for compliance (see Martela et al., 2021) were more pronounced at the beginning of the outbreak. For instance, people were more afraid as the perceived risk of being infected or suffering severe consequences from the disease were higher than a year later (T2). On this regard, research have shown that both fear (Christner et al., 2020; Pakpour & Griffiths, 2020) and risk perception (de Bruin & Bennett, 2020; Schmitz, Luminet, et al., 2022; Siegrist et al., 2021) enhance the enactment of protective measures. Also, the health measures were more stringent (e.g. stay at home was mandatory, sanitizing hands when entering or leaving public spaces), and the external contingencies (e.g. social pressures, fines) more pronounced at the beginning of the crisis than later on. Interestingly, the change in the mean level of handwashing across time remained almost unchanged despite the considerable mean level decrease in the TPB-intentions and TPB-norms predictors.

It is important to note however that the resilience to comply with this health behavior might not generalize to other more costly and ‘unnatural’ behaviors such as social contact limitation which runs counter to the very social nature of humans being and has proven to be detrimental to mental health and livelihood (Hagger et al., 2020; Pogrebna & Kharlamov, 2020; West et al., 2020). To illustrate, Wollast et al. (2022) found that participants belonging to trajectory groups with high levels of social contacts limitation and physical distancing experienced poorer mental health, but this was not the case for participants having high levels of adherence to handwashing over time.

Along these lines, a second noteworthy finding concerns the relative stability in the prediction pattern across time. Indeed, being part of the (para)medical field, being angry/agitated, together with two TPB components, namely intentions and perceived control fostered compliance with handwashing at both T1 and T2, with the later time point having the largest effect size. Nonetheless, slight discrepancies between predictors emerged across time, such that being a female had only a positive effect at T1, and social norms fostered the behavior only at T2. However, the effect sizes of these factors were quite small. A plausible explanation for the emergence of the social norms at T2 (but not at T1) could be accounted by the fact that social norms usually develop gradually over time and because they require direct social interactions to be internalized, which were prohibited during the first lockdown of the pandemic (Rimal & Storey, 2020). Also, and in line with Bigot et al. (2021), neither impulsivity nor social connection components yield significant effects, suggesting that this behavior may not require inhibitory processes to be enacted and may not be facilitated by one’s social network. In this regard, handwashing contrasts with other health behaviors such as social contacts limitation which does run counter to human basic needs (i.e. socialization; and may thus require inhibitory processes) and could be more directly affect by social connection. As a set these second finding goes thus hand in hand with the first one, as it may suggest that the stability of the adherence level may stem from the remarkable stability of its underlying factors.

A key theoretical implication from the above results is that it highlights the robustness of the TPB. Indeed, this model has been successfully used to predict many other health behaviors that do not entail such detrimental mental health consequences (e.g. healthy diet consumption; Lin & Roberts, 2020). Further research is therefore advised to clarify if the (lack of) compliance with more costly behaviors such as social contact limitation would be accounted for by the TPB. More generally, the current investigation enriches the literature by further identifying and understanding which are the unique and most important factors that undermine or promote the adherence to this specific and widely recommended health behavior.

The present findings also have valuable practical implications to help overcome the ongoing crisis, in particular for policy makers or communication campaigns. As a set, our results convey the message that the same strategies could be used to promote this health behavior given the high stability of its underlying factors. For instance, handwashing could be boosted by promoting perceived behavioral control (e.g. through ‘nudging techniques’, Broers et al., 2019a, 2019b) throughout the pandemic. Moreover, the stable level of adherence to handwashing despite varying circumstances suggests that, if needed, resources may be allocated towards promoting other health behaviors that tend to be harder to sustain.

A limitation from the present research is that only participants that completed both surveys (at T1 and T2) were considered in the analyzes. Another limitation is that the health behavior was measured by the mean of a single item. Future research could improve this measurement by incorporating several items that capture the various aspects of these behaviors. As a case in point, Wollast et al. (2022) overcame this limitation and validated new measures by capturing numerous facets of each health behavior (e.g. alone vs. with others) in many different social situations (e.g. private or public places, public transports, at home) by relying on multiple items for handwashing, mask wearing, social contacts limitation, and physical distancing. The prospective analysis successfully replicating the main model on handwashing measured at T2, while controlling for T1 handwashing, is a first step in the direction of respecting the causal ordering of the variables. Nevertheless, this evidence is not sufficient to establish causal relations. To characterize patterns of variation in handwashing over time, we encourage scholars to rely on a latent class growth analysis to identify homogeneous subgroups of handwashing within the larger heterogeneous population (see for example Wollast et al., 2022). Finally, generalization of the present findings should be taken with caution given the relatively small dataset.

In conclusion, our findings highlight the stability of handwashing and its associated predictors over time, across countries, and under distinct circumstances that may be more or less critical.

## Notes

1 We relied on these two countries as they went through similar pandemic-related policies restrictions.

2 Although the authors collected data from different French-speaking countries in Europe, their work was focused only on the Belgian sample.

3 We report Cronbach’s *α* for T1 given that there is a bug in the MBESS R package for that specific timepoint (see https://github.com/Matherion/userfriendlyscience/issues/14).

4 Linear Mixed-Effects Models were initially used to assess the effect of time (T1 vs. T2), both as a predictor and as an interaction term with the other predictors. However, none of the models converged to a stable solution.

5 We did not use multiple tests comparison adjustments (e.g. Bonferroni) given that we did not perform multiple statistical tests on the same dependent variable.
